# Simvastatin as an immunomodulator and anti-myeloma agent – epidemiological, *in vitro* and murine model studies

**DOI:** 10.3389/fimmu.2026.1882291

**Published:** 2026-08-19

**Authors:** Aharon Lion, Arieh Y. Israel, Eugene Merzon, Howard Oster, Abraham Weizman, Galia Luboshits, Bjarne Bogen, Moshe Mittelman, Michael A. Firer

**Affiliations:** 1 Department of Chemical Engineering, Ariel University, Ariel, Israel; 2 Leumit Health Services, Tel Aviv-Yafo, Israel; 3 Department of Hematology, Tel-Aviv Sourasky Medical Center and Tel-Aviv University Medical School, Tel Aviv, Israel; 4 Department of Medicine, Tel-Aviv Sourasky Medical Center and Tel-Aviv University Medical School, Tel Aviv, Israel; 5 Research Unit, Geha Mental Health Center, Felsenstein Medical Research Center and Sagol School of Neurosciences, Gray Faculty of Medical and Health Sciences, Tel-Aviv University, Tel-Aviv, Israel; 6 Department of Immunology, Institute of Clinical Medicine, University of Oslo and Oslo University Hospital, Oslo, Norway; 7 Adelson School of Medicine, Ariel University, Ariel, Israel

**Keywords:** multiple myeloma, non-prescription drugs, prevention, repurpose, Simvastatin

## Abstract

**Introduction:**

Simvastatin (SIM), a member of the statin family, is commonly used to lower blood cholesterol levels, particularly low-density lipoprotein. Statins inhibit the action of hydroxy-methyl-glutaryl-coenzyme A reductase, an important enzyme in the synthesis of cholesterol and isoprenoids; they also have anti-inflammatory effects and can regulate membrane synthesis in cancer cells. Preliminary evidence suggests an association between SIM use and reduced cancer incidence. SIM’s anti-cancer efficacy *in vivo* has been shown in studies on several solid tumors, but little is known about its effects on hematological cancers, such as Multiple Myeloma (MM).

**Methods:**

Initially, we searched for such as an association in a large community database and observed a significantly lower MM incidence among SIM users. We proceeded to test the effect of SIM on both murine and human MM cell lines and the tested the effect of long-term SIM treatment on development of MM in an animal model.

**Results:**

SIM exposure led to apoptosis in both murine and human MM cell lines. We show that long-term intake of SIM reduced tumor load and positively impacted bone marrow CD8+ T levels as well as cell-mediated anti-myeloma cytotoxicity. SIM treatment also elevated the total IgA and IgG serum concentrations in healthy mice.

**Conclusions:**

These results suggest that SIM, which many adults use to control cholesterol levels, may play a dual role in the management of MM, by influencing membrane synthesis in myeloma cells leading to apoptotic cell death and enhancing the T-cell mediated anti-tumor cell response in MM.

## 1 Introduction

Advances in molecular and cell biology, along with the skyrocketing costs of cancer drug development, are driving research into the repurposing of existing drugs, including those initially intended for non-cancerous conditions. This strategy is predicted to lead to quicker and more affordable progress in cancer treatments, as these drugs have already completed regulatory approval processes and are currently in clinical use. Since the safety profile is known, their use might save time and the cost of at least Phase I clinical trials. In the case of Multiple Myeloma (MM), a classic example of drug repurposing is Dexamethasone, which has been a foundational, “repurposed” treatment for multiple myeloma (MM) for over 50 years, transitioning from a general anti-inflammatory steroid to a key anti-myeloma agent (1).

One drug studied intensively for its off-target effects is Simvastatin (SIM), a member of the statin family commonly used to lower blood cholesterol levels, particularly low-density lipoprotein cholesterol. In 2012, a CDC study found that in the US alone, 42% of adults over the age of 40 were regularly using prescription SIM (https://www.cdc.gov/nchs/products/databriefs/db177.htm). Several investigations have evidenced the positive epidemiological correlation between the long-term use of SIM and cancer survival (2–4). Statins in general inhibit the action of hydroxymethylglutaryl-coenzyme A reductase, an important enzyme in the mevalonate pathway (5) responsible for synthesizing cholesterol and isoprenoids. These processes are also critical for the proliferation and survival of cancer cells (2, 6, 7), and recent studies have identified cholesterol homeostasis as a key factor in cancer pathogenesis (8). Additionally, SIM can enhance T cell activation (9), the reprogramming of lymphocytes and macrophages (10), and stimulate the secretion of inflammatory cytokines (9).

SIM’s anti-cancer efficacy *in vivo* has been shown in studies on several solid tumors (11, 12), but there is limited information regarding the effect of SIM or other non-cancer prescription drugs on hematological cancers. Given the need to identify new measures that may prevent or at least delay the onset of these malignancies, we began the present study by conducting an epidemiological analysis to search for correlations between the regular use of non-cancer prescription drugs, and the incidence of MM in the Israeli population. MM is a malignant plasma cell tumor, and the second most common hematological disease, after non-Hodgkin’s lymphoma. The disease is characterized by the proliferation of abnormal “long-living” plasma cells in the bone marrow that disrupt hematopoiesis, leading to anemia, bone lesions, hypercalcemia, and kidney damage, neuropathy, and coagulopathy (13). Despite recent advances in treatment methods (14, 15), MM remains essentially incurable, posing significant challenges to patients and health-care systems. Therefore, new therapeutic approaches such as drug repurposing that might be efficacious in halting or delaying the progression of MM should be explored. Since 5-20% of the adult Israeli population uses statins, including SIM, for their prescribed purpose (which aligns with rates in other developed Western countries https://www.singlecare.com/blog/news/statin-statistics/), we tested whether long-term SIM use influenced the incidence of MM in the Israel population. Using the Leumit Health Systems (LHS) electronic databases, which contain detailed clinical and demographic data on 730,000 Israelis from a cross-section of ethnic backgrounds, we found a striking decrease in the incidence in MM amongst LHS clients on long-term consumption of SIM. Based on these results we then carried out *in vitro* studies, demonstrating the effect of the drug on MM cell lines. Subsequent studies in an immunocompetent mouse model of MM showed that long-term intake of SIM inhibited disease progression and modulated the anti-tumor immune response.

## 2 Materials and methods

### 2.1 Epidemiology of non-cancer prescription drugs and the incidence of multiple myeloma

A retrospective matched case-control study was conducted using the Leumit Health Services (LHS) electronic health record database, which includes longitudinal clinical, laboratory, pharmacy purchase, and demographic data. We first identified patients diagnosed with MM. The diagnosis was made in a university hematology clinic. Patients were included if they had at least two years of documented membership prior to the index date.

A total of 724 MM patients were identified and matched to 14,480 controls (1:20 matching) on birth year, sex, socioeconomic status, smoking status, and first year of LHS membership. Controls were assigned the same index date as their matched case. For each individual, medication purchases of non-chemotherapy prescription drugs were extracted for up to 7 years prior to the index date. Drug exposures were defined as at least one purchase during the period.

A screening analysis was conducted to identify medications that were significantly less frequently used among MM cases compared to controls, using Fisher’s exact test. Statistical analyses were performed with R 4.3.

### 2.2 Cell culture

The murine BM-homing myeloma cell line MOPC315.BM secreting an IgA isotype paraprotein with lambda light chains was generously provided by Prof. Bjarne Bogen (University Hospital Oslo, Norway), and grown at 37 °C in 5% carbon dioxide in RPMI 1640 medium (Sigma-Aldrich, Israel) supplemented with 10% FBS (Biological Industries, Bet Haameck, Israel), 1% MEM NEAA 100x (Gibco, Israel), 0.005% 1M I-thioglycerol, 40 mg/ml 0.03% Gensumycin (Sigma Aldrich), and 2 mM L-glutamine (Sartorius). The human myeloma cell lines, RPMI-8226 and U-266BI were purchased from the ATCC and grown at 37 °C in 5% carbon dioxide in RPMI 1640 medium supplemented with 15% FBS, 1% Pen-Strep (Gibco), and 1% L-glutamine. SIM (Combi-Blocks, CA, USA) was dissolved in DMSO and the stock solution maintained at 4 °C. For *in vitro* and *ex vivo* experiments, the stock solution was diluted in appropriate growth medium. For *in vivo* studies, the drug was dissolved in a 1:1 DMSO: Tween20 solution and diluted in PBS.

### 2.3 Cytotoxicity test

To test the cytotoxic effect of SIM, 1.5 x 10^5^ of MOPC315.BM, RPMI-8226 or U-266BI cells/ml were seeded into 96 well microplates (Nunc, Thermo Fisher, MA, USA) alone or together with SIM in concentrations ranging from 1.5 nM to 50μM. Due to differences in cell growth rate between the cell lines, MOPC315.BMcells and RPMI-8226 were cultured with drug for 72 hours, while U-266BI cells were incubated for 96 hours. Cellular metabolism was taken as a measure of cell viability and was assessed by the addition of XTT or Alamar Blue reagent (Sartorius, Gottingen Germany). Optical Density (OD) of XTT was measured at 450nm, with a reference measurement at 630nm) reagent, while the OD of Alamar Blue was measured at 570nm, with a reference measurement at 600nm. Experiments were performed three times, each time in triplicate. The ODs were measured using a TECAN Infinite M200 reader, and used to calculate the % Growth Inhibitory (%GI) for each cell line. The resultant dose-response curves were used to calculate the IC50 values.

### 2.4 Mechanism of cell death

To determine if SIM induced apoptosis, cells were seeded and cultured as described above together with SIM at the appropriate IC50 concentration. At the end of the culture period, cells were recovered and tested for apoptosis with the Apoptosis Detection Kit (ab214484, Abcam) according to the manufacturer’s instructions using a Cytoflex flow cytometer. Appropriate controls were included with which to perform gating and compensation standardization.

### 2.5 Cell cycle analysis

The effect of SIM on the cell cycle was examined by seeding cells into 6-well plates and culturing them as above with SIM. Cells were then washed, resuspended at a concentration of 10^6^ cells/ml, stained with 20 μM Hoechst 33342 (Thermo Fisher) or 75μM Propidium Iodide. RNase (ab139418, Abcam) was added and the mixtures incubated at 37 °C for 30 minutes and analyzed by flow cytometry. The FSC, SSC and staining distributions were analyzed with FlowJo V10.8.1 software to determine the percentage of cells in each stage of the cell cycle. The distributions of the treated groups were compared to the corresponding control cells by performing a Graphpad-assisted Fisher’s Exact test.

### 2.6 Effect of SIM on an animal model of early-to-late MM

All animal experiments were performed according to the protocol approved by the Institutional Committee for the Treatment and Use of Animals at Ariel University (AU-IL-2401-103). The recently reported ELMM mouse model (16–18), which portrays the serological and immunological features of human MM, was used to test the effect of SIM on the progression of MM disease and changes in immune profile over an extended period.

Prior to inoculation, fifteen-week-old male Balb/c mice (Envigo Laboratories, Jerusalem, Israel) were randomly assigned to one of four groups. Group 1 mice were treated with vehicle alone (NoSIM/NoMM), Group 2 received SIM treatment (SIM/NoMM), Group 3 mice were myeloma-bearing and were treated with vehicle (NoSIM/MM), and Group 4 mice were myeloma-bearing and received SIM treatment (SIM/MM). NoSIM/MM and SIM/MM mice were inoculated into the tail vein with 5,000 MOPC315.BM cells, while NoSIM/NoMM and SIM/NoMM mice received vehicle alone. Following inoculation, mice were treated by intraperitoneal (I.P.) injection three times a week for 21 weeks with either vehicle alone (200μL of PBS: DMSO: Tween 20 at a ratio of 90:5:5), or 23mg/Kg of SIM dissolved in vehicle. This SIM dose is equivalent to the daily recommended dose for humans of 40 mg (19, 20). Blood samples were collected from the tail vein of all mice on the day of inoculation (day 0) and serum was stored at -20^0^C until used. In our previous report describing the ELMM model (18), we analyzed samples at three time points representing the early, mid and later stages of myeloma progression. Therefore, in the present study, at least five mice from each group were sacrificed on days 65 (early stage) and 190 (mid to late stage) after inoculation. Cardiac blood was recovered, and the serum was stored at -20^0^C until used. BM aspirates were recovered by flushing the femurs and tibias with PBS. Following removal of red cells by addition of RBC Lysis buffer (eBioscience) and centrifugation, single cell BM suspensions were subjected to immune profiling and T cell functional tests as described below.

### 2.7 ELISA for serum paraprotein

To measure serum paraprotein levels, Nunclon ELISA microplates were coated with 5μg/mL goat anti-mouse IgA (Southern Biotech) overnight at 4 °C. The wells were blocked with PBS/0.02% sodium azide/1% BSA, washed and incubated for 1 hour at 37 °C with serum samples (diluted to 1:4000) or with purified mouse IgA (diluted to 3.75ng - 625ng/mL), diluted in PBS/0.02% sodium azide/0.1% BSA/0.1% Tween 20. The plates were then washed and incubated with goat anti-mouse IgA Fc HRP (Southern Biotech) diluted to 1:40,000 for 45 minutes at room temperature and washed again. TMB Substrate (Merck Millipore, Billerica) was added for 10 minutes and the reaction stopped by the addition of 3M H_2_SO_4_. Absorption was measured at 450nm using the TECAN Infinite M200 ELISA reader.

### 2.8 Immune profiling of myeloma bone marrow aspirates

The effect of SIM treatment on the immune response as MM developed was monitored by flow cytometric immune profiling of BM aspirates recovered as described above. Single cell BM suspensions were Fc receptor blocked by incubation with monoclonal mouse IgG anti-CD16/CD32 (eBioscience clone 93). Each sample was then stained for 30 min at RT in the dark with combinations of fluorescently labeled anti-CD marker antibodies selected to analyze the frequency of CD4+ and CD8+ T cell subsets and MOPC315.BM (B220-/CD138+) (see **Supplementary Tables 1**, **2**). Fluorescence was measured with a Cytoflex (eBiosciences) flow cytometer, and the data were analyzed with FlowJo software (TreeStar, Ashland, OR, USA). The gating strategy and compensation matrix for each panel included FMO controls. Gating schemes and antibodies are shown in **Supplementary Tables 1**, **2** and **Supplementary Figure 1**.

### 2.9 *In vitro* T cell activation and cytotoxicity assay

MOPC315.BM cells were cultured for 24 hours in complete medium (RPMI 1640, 10% FBS, 1% Penicillin/Streptomycin, 2 mM L-glutamine, and 50 μg/mL 2-mercaptoethanol) containing 25μg/mL mitomycin C (Sigma) to halt cell division. Then, for each well of a 6-well plate, 5 x 10^6^ BM cells were mixed with the mitomycin-treated MOPC315.BM target cells at a ratio of 20:1 (BM: MOPC) and cultured for 2–4 days in complete medium supplemented with 5μg/mL anti-CD3 antibodies, 2μg/mL anti-CD28 antibodies and 50U/mL recombinant IL-2 (BioLegend) (21). From these cultures, CD8-positive T cells were isolated using magnetic microspheres (Miltenyi, Bergisch Gladbach, Germany) according to the manufacturer’s instructions. The purity of the isolated CD8+ T cells was verified by flow cytometry. To assess T-cell cytotoxicity, 10^7^ fresh MOPC315.BM cells were first labeled with carboxyfluorescein succinimidyl ester (CFSE) (eBioscience) at a concentration of 1μM for 10 minutes at room temperature. The reaction was stopped by adding four volumes of cold complete medium and incubating on ice for 5 minutes. After washing with complete medium, the labeled MOPC315.BM cells were suspended in complete medium at a concentration of 10^6^ cells/mL and transferred to wells in a 96-well plate (100 μL/well). The isolated CD8-positive T cells were then added to the target cells at a ratio of 20:1 (T cells:MOPC315.BM) in a final volume of 250 μL/well and the cell mixture was cultured for 4 hours. To distinguish between live and dead cells, the LIVE/DEAD™ Fixable Red Dead Cell Stain kit (Thermofisher Scientific) was used. Target cells incubated without T cells served as controls for spontaneous cell death. Fluorescence data were collected using a flow cytometer and analyzed with FlowJo v10.8.1 software. The percentage of MM cell killing was calculated with the formula:


% MM cell death=(% CFSE MM cells alone − % CFSE MM cells with effector cells)/(% CFSE MM cells alone)

### 2.10 Statistics

Statistical analyses were performed using Excel and GraphPad Prism. IC50 values ​​induced by SIM were calculated using non-linear regression. Differences between the IC50 values ​​of the different groups were examined using the Extra sum-of-squares F-test. The cell cycle analysis was performed using a chi-squared test. For *in vivo* experiments, power and sample size required to reach a significance level of α= 0.05 between groups were calculated using PS software from Vanderbilt University. The flow cytometry data on immune cell populations were analyzed using FlowJo V10.8.1 software, applying a hierarchical gating strategy to identify and quantify specific cell populations. Statistical differences in immune cell frequencies between groups were tested by two-way ANOVA or multiple-groups analysis with Bonferroni’s adjustment using Graphpad 10.6.1 software.

## 3 Results

### 3.1 Long-term consumption of SIM correlates with decreased incidence of MM – human epidemiological studies

In the LHS matched cohort (N = 724 MM patients, N = 14,480 controls), the mean age at the index date was 66.9 ± 11.9 years, with 52.3% male participants in both groups. Data mining revealed the prescription drug purchase history of patients and controls during the seven years preceding the index date. Using Fisher’s rigorous testing, we found that any recorded use of SIM during the seven years preceding the index date was significantly associated with a reduced risk of developing MM, as seen in **Table 1**, which describes the positive associations between prescription drug purchases in the seven years preceding the index date and the decrease in the chance of developing MM using odds ratio and statistical significance. These epidemiological data are suggestive that the drug may play a role in preventing/delaying the progression of MM.

**Table 1:** Statistical relationship between the use of prescription Simvastatin purchase during the 7 preceding years the index date and the diagnosis of clinical myeloma.

Drug	% usage in MM group	% usage in control group	Odds ratio of developing MM	P- value
Simvastatin	44.06 (N = 724)	49.66 (N = 14,480)	0.798389	0.003349

### 3.1 SIM is directly cytotoxic to MM cell lines

**Figure 1:**
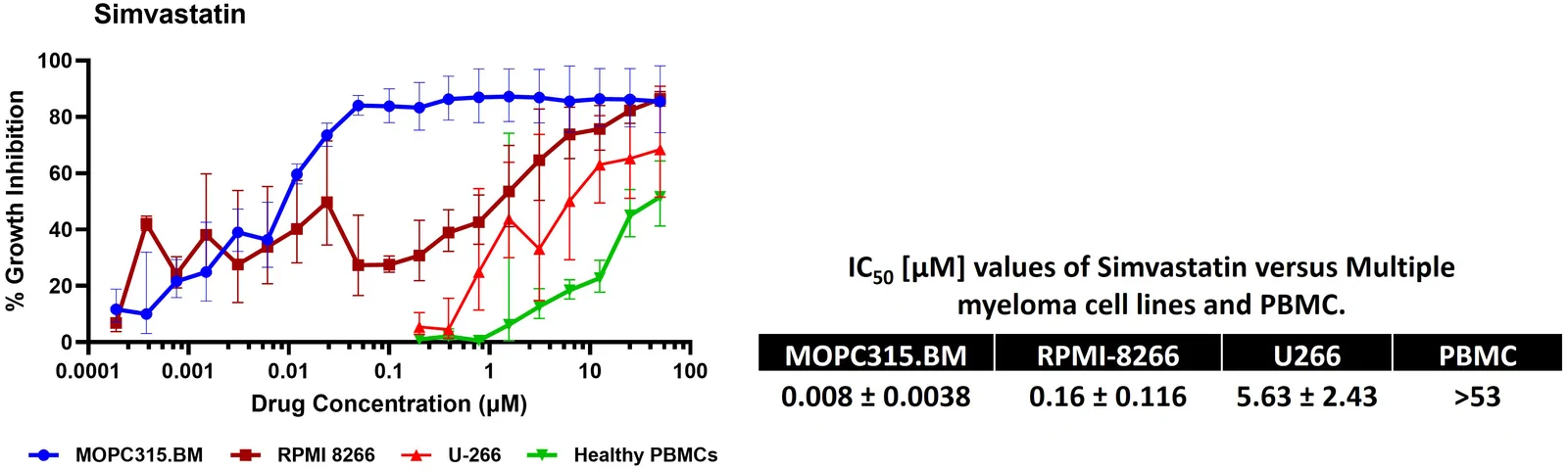
Dose-response curves of SIM on the growth of myeloma cell lines. Data points show the percent cell growth inhibition as calculated relative to cells grown without drug. Each point represents the mean ± standard error of at least three experiments each run in triplicate. MOPC315.BM, RPMI-8226, U-266BI, and Peripheral Bloon Mononuclear Cells (PBMCs).

Murine MOPC315.BM and human RPMI-8226 cells and U-266BI cells were cultured in the presence or absence of increasing doses of SIM. The percentage of growth inhibition at each drug dose (%GI) was calculated using cells grown without drug as 0%GI control. The resulting dose-response curves were used to calculate IC50 values against each cell line. **Figure 1** shows clear heterogeneity in the cytotoxicity of SIM against murine or human MM cell lines. Murine MOPC315.BM cells are highly sensitive to SIM with an IC50 of 0.008µM, compared to the IC50 values of 0.1638 and 5.637µM for RPMI8266 and U-266 respectively. The IC50 of SIM versus healthy human Peripheral Blood Mononuclear Cell (PBMCs) was >53µM.

### 3.2 Mechanism of SIM cytotoxicity

**Figure 2:**
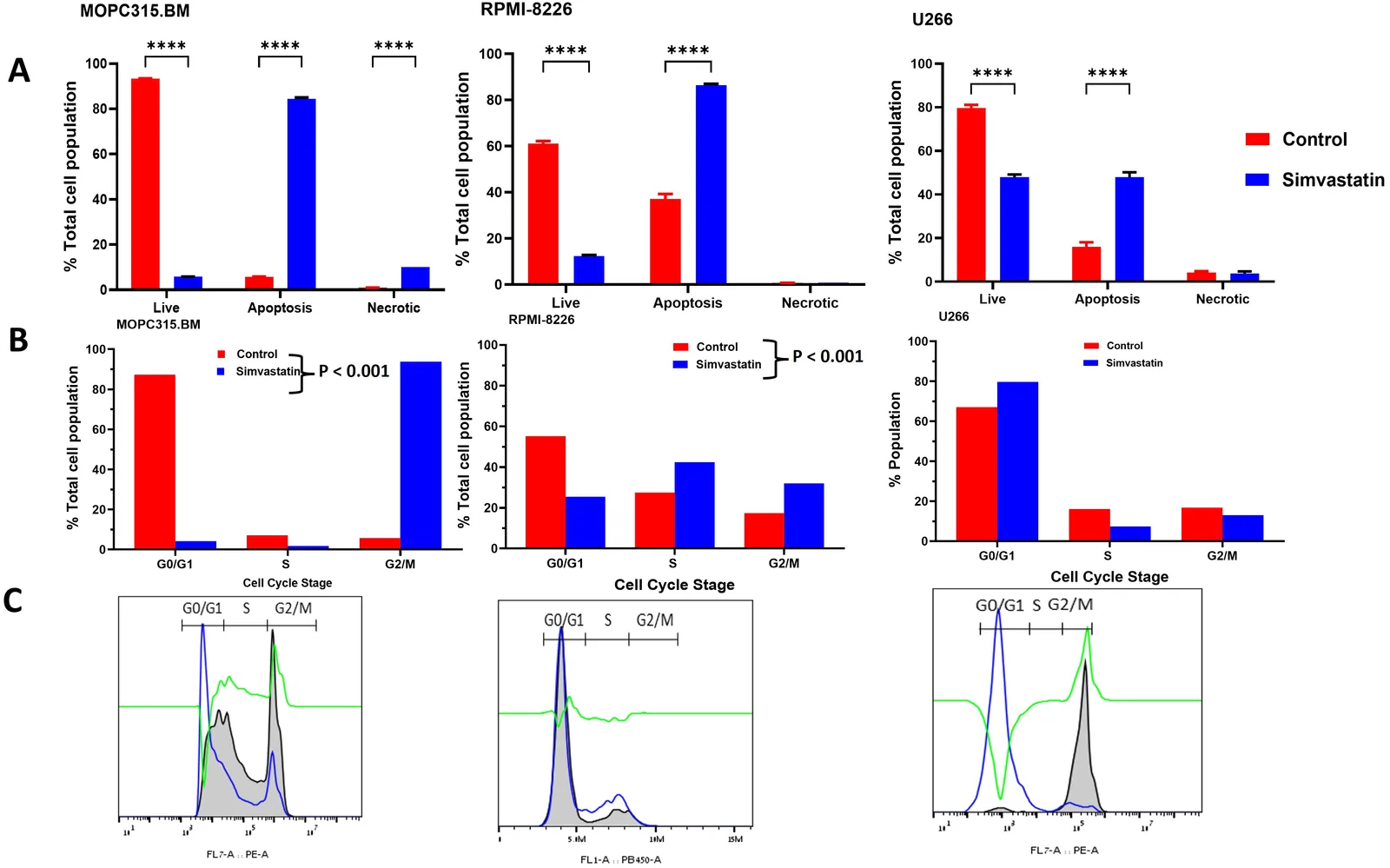
**(A)** Effect of Simvastatin on myeloma cell death by apoptosis or necrosis. Murine (MOPC315.BM) and human (RPMI-8266 and U266B1) myeloma cells were exposed to the drug at the IC50 dose for 48hrs and then assayed by flow cytometry for expression of Annexin V expression and PI uptake. **(B, C)** Effect of Simvastatin on the cell cycle on the same myeloma cells. Cells were exposed to the IC50 dose of the drug for 48hrs, stained with 20 μM Hoechst 33342 and assessed for DNA content by flow cytometry. Statistical differences between groups were determined by two-way ANOVA. **p<0.01, ***p<0.001, ****p<0.0001.

The pathway of cell death resulting from the cytotoxic effect of SIM was examined by exposing cells to the IC50 dose and measuring the binding of Annexin V and PI uptake of cells as markers of cells undergoing apoptosis or necrotic death, respectively. **Figure 2A** demonstrates that SIM induced apoptosis in all three cell lines (p <0.0001 for all cases).

### 3.3 The effect of the drugs on the cell cycle

Myeloma cell lines stained with Hoechst 33342 or PI were incubated with or without IC50 concentrations of SIM and analyzed for fluorescence by flow cytometer. **Figures 2B, C**, show that SIM stalled the MOPC315.BM cells in G2/M, (p<0.001, Fisher’s exact test). Similarly, SIM significantly altered the cell cycle distribution of RPMI-8226 cells (p<0.001, Fisher’s exact test), but the drug did not significantly affect the cell cycle of U266 cells.

### 3.4 Influence of Simvastatin on the immune response in a murine model of MM

**Figure 3:**
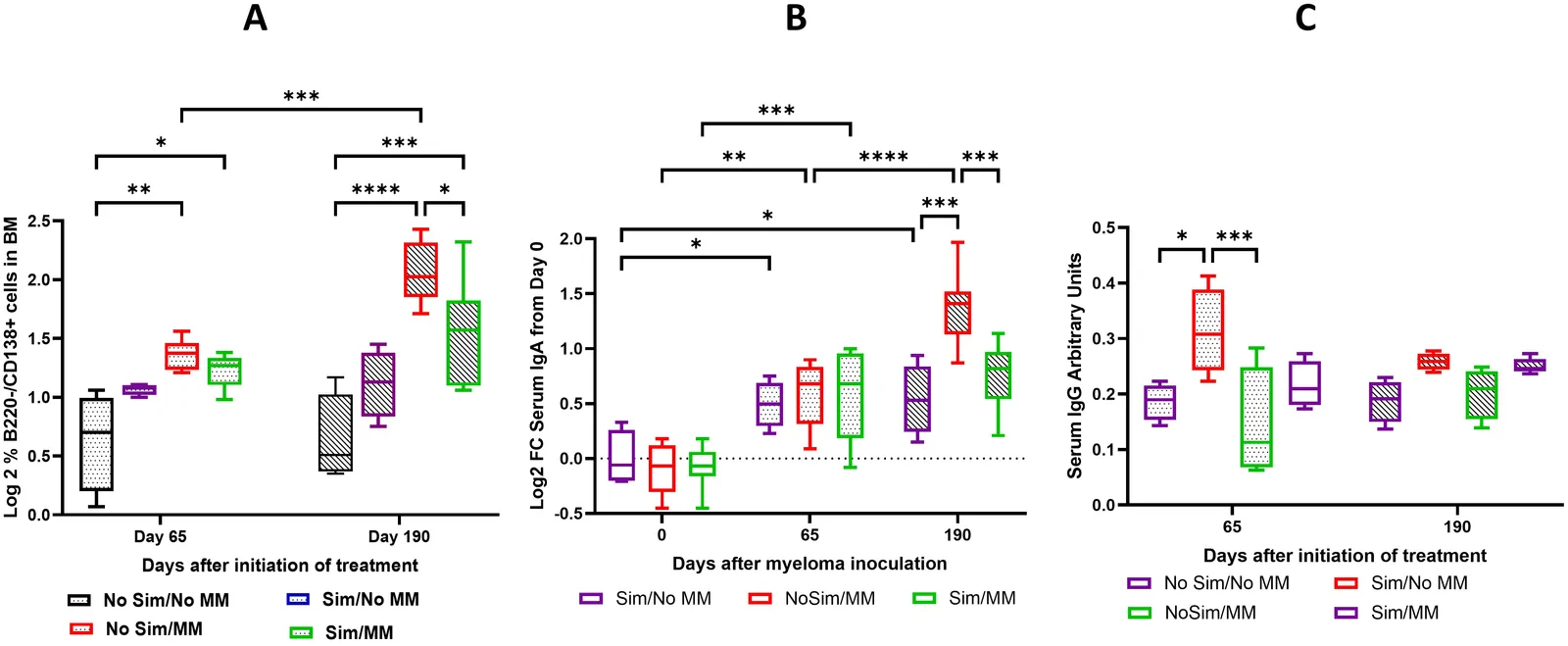
**(A)** Log2 transformed percentage of MOPC315.BM (B220-/CD138+) myeloma cells in bone marrow cell suspensions 65- and 190-days post-inoculation of Simvastatin-treated or non-treated Balb/c mice. The myeloma cells were identified as B220-/CD138+ by flow cytometry. **(B)** Log2 Fold Change in total serum IgA levels (µg/ml) in the three groups of mice 65- and 190-days post-myeloma cell inoculation of Simvastatin-treated or non-treated Balb/c mice. **(C)** Comparison of total serum IgG levels (in Arbitrary Units) of all mice 65- and 190-days post-myeloma cell inoculation. Significant differences between groups were assessed using a two-way ANOVA in Graphpad Prism version 10.6.1. *p<0.05; **p<0.01, ***p<0.001, ***p<0.0001, ****p<0.00001. N.B: Only significant differences mentioned in the text are shown. For each group, at each time point n(no. of animals) ≥ 5.

Based on the above results, we asked whether sustained treatment with Simvastatin influences growth of MOPC315.BM cells as well as immune profiles and lymphocyte effector functions *in vivo*. For this purpose, we utilized our long-term (ELMM) mouse model of MM (18). On Day 65 and 190 following inoculations with 5000 MOPC315.BM cells, blood samples were collected for measurement of serum IgA, and BM samples were immune profiled by flow cytometry (see **Supplementary Information**) for MOPC315.BM cells (B220-/CD138+) and the presence of CD4+ and CD8+ T cells within the CD3+ population. **Figure 3A** depicts the development of myeloma disease in non-treated mice with a significant and continual rise over time in the frequency of MOPC315.BM cells in BM, accompanied by significantly elevated total serum IgA due to the production of IgA paraprotein by the MOPC315.BM myeloma cells (**Figure 3B**). Simvastatin-treatment reduced the amounts of MM cells in BM both at day 65 and 190 although the reduction was only significant at day 190 (**Figure 3A**). Consistent with this, Simvastatin-treated mice had significantly reduced amounts of IgA M315 myeloma protein in sera at day 190. (**Figure 3B**). Interestingly, IgA levels were increased above baseline in the SIM/NoMM group at Day 65 and remained so at Day 190 (**Figure 3B**). The enhancing effect of SIM was not IgA-specific since we found that serum IgG levels in these mice were also significantly increased at Day 65 and remained above control levels on Day 190 (**Figure 3C**). Thus, SIM treatment nonspecifically increased levels of several Ig classes.

**Figure 4:**
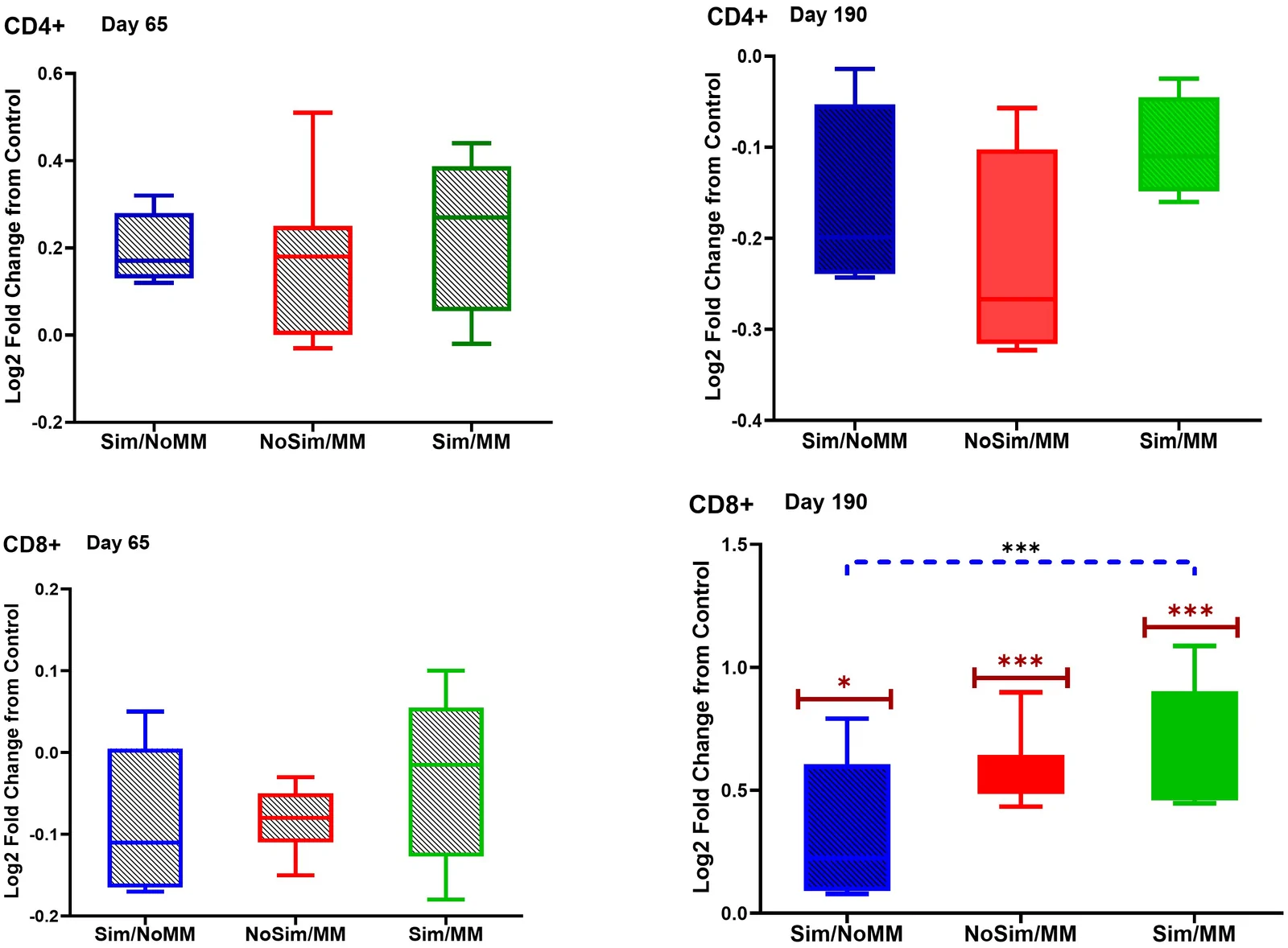
Effect of long-term Simvastatin treatment on changes in T cell frequencies in the bone marrow of mice in the Early-to-Late model of MM. Balb/c mice were inoculated i.v. with 5000 MOPC315.BM cells on Day 0 and given SIM twice weekly as described in Materials and Methods. On Days 65 and 190, bone marrow mononuclear cells were profiled by flow cytometry for the frequencies of CD4+ and CD8+ cells within the CD3+ population. % frequencies were Log2 transformed and the significance of Fold Changes (FC) in the test groups (SIM/NoMM, NoSIM/MM and SIM/MM) compared to control mice (NoSIM/NoMM), as well as between the various test groups, were analyzed by Mixed effects analysis or 2-way ANOVA with Bonferroni’s test, performed in GraphPad version 10.6. Brown solid bars represent statistically significant differences between the test groups and the controls (above = increase). Blue dashed bars represent differences between the test groups. *p ≤ 0.05-0.01; **p ≤ 0.001; ***p ≤ 0.0001. For each group, at each time point n(no. of animals) ≥ 5.

**Figure 4** shows the Log2 Fold Change (FC) in T cell frequencies in the three test groups over the average frequency of vehicle treated controls (NoSIM/NoMM). For CD4+ T cells, there were no differences among the groups at Day 65, while levels in the vehicle treated NoSIM/MM group tended to be lower than those in the SIM/MM group at Day 190. In contrast, while the levels of CD8+ cells in the three test groups were similar to control mice at Day 65, by Day 190 the FCs in CD8+ cells in SIM/NoMM mice were significantly higher than controls (**Figure 4**, p<0.001). CD8+ levels in the SIM/MM group tended to be higher than those in the NoSIM/MM group, but this difference did not reach statistical significance. However, the increase was significantly greater in SIM/MM than in SIM/NoMM mice (blue dashed line, p=0.001).

**Figure 5:**
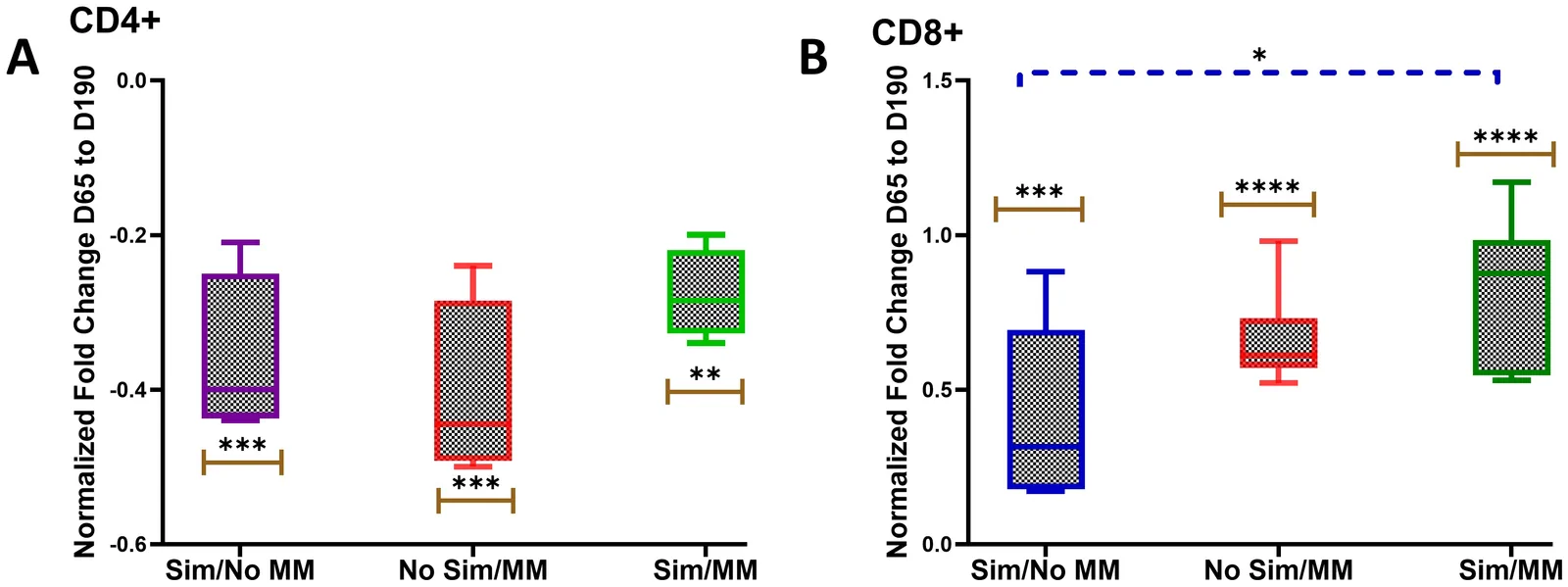
Effect of long-term Simvastatin treatment on changes over time in T cell frequencies in the bone marrow of mice in the Early-to-Late model of MM. Balb/c mice were inoculated i.v. with 5000 MOPC315.BM cells on Day 0 and given SIM twice weekly as described in Materials and Methods. On Days 65 and 190, bone marrow mononuclear cells were profiled by flow cytometry for the frequencies of CD4+ **(A)** and CD8+ cells **(B)** within the CD3+ population. % frequencies were Log2 transformed, and the Fold Changes (FC) in the test groups (SIM/NoMM, NoSIM/MM, and SIM/MM) from control mice (NoSIM/NoMM) were determined. The significance of changes in the normalized FC for each test group as well as between the test groups from Day 65 to Day 190 were analyzed by 2-way or Ordinary ANOVA analysis with Bonferroni’s test, performed in GraphPad version 10.6. Brown solid bars represent statistically significant differences between the two time points (above the box = increase, below the box = decrease). Blue dashed bars represent differences between the test groups. *p ≤ 0.05-0.01; **p ≤ 0.001; ***p ≤ 0.0001. For each group, at each time point n (no. of animals) ≥ 5.

We next analyzed changes in these immune profiles over the two time periods by comparing the FC of each test group on Days 65 and 190. As suggested by the results in **Figure 4**, the levels of CD4+ cells normalized to controls decreased significantly in all three test groups between Days 65 and 190 (**Figure 5A**), while the normalized FC changes in CD8+ frequencies increased dramatically in all three test groups (**Figure 5B**). This increase was significantly greater in SIM/MM mice than in SIM/NoMM mice (p=0.0233).

**Figure 6:**
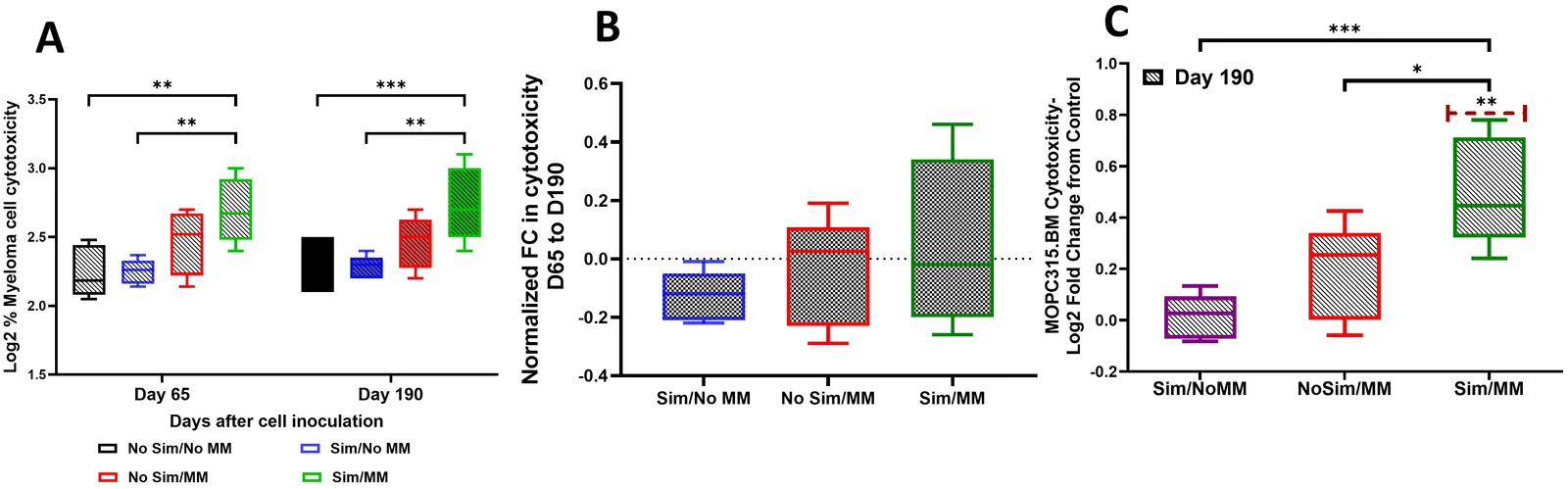
Effect of long-term Simvastatin treatment on the anti-myeloma cytotoxicity of CD8+ T cells in the bone marrow of mice in the Early-to-Late model of MM. On Days 65 and 190 after inoculation of mice with vehicle or MOPC315.BM myeloma cells, CD8+ T cells were isolated from bone marrow mononuclear cells of the mice and cultured for 4 hrs with fresh CFSE-labeled MOPC315.BM cells at a ratio of 20:1 (T cells:MOPC315.BM). Target cells incubated without T cells served as controls for spontaneous cell death. Fluorescence data were collected using a flow cytometer and analyzed by FlowJo v10.8.1 software. The percentage of MM cell killing was calculated with the formula: % MM cell death= (% CFSE MM cells alone - % CFSE MM cells with effector cells)/(% CFSE MM cells alone) **(A)**. The significance of changes in the normalized FC for each test group as well as between the test groups from Day 65 to Day 190 **(B, C)** were analyzed by 2-way or Ordinary ANOVA analysis with Bonferroni’s test, performed in GraphPad version 10.6. *p ≤ 0.05-0.01; **p ≤ 0.001; ***p ≤ 0.0001.

Long-term exposure to SIM also affected the cytotoxic function of CD8+ T cells. Only SIM/MM mice showed a significant increase in myeloma cell killing above NoSIM/NoMM and SIM/NoMM mice, both at day 65 and day 190 (day 65 p <0.01, p<0.01 and day190 p<0.001and p<0.01 respectively) (**Figure 6A**). Furthermore, by day 190, Log2 fold changes above controls were again only significantly greater in SIM/MM mice (p<0.001) and importantly were also significantly greater than in NoSIM/MM mice (p<0.02).

### 3.5 Simvastatin consumption delays development of active myeloma

**Figure 7:**
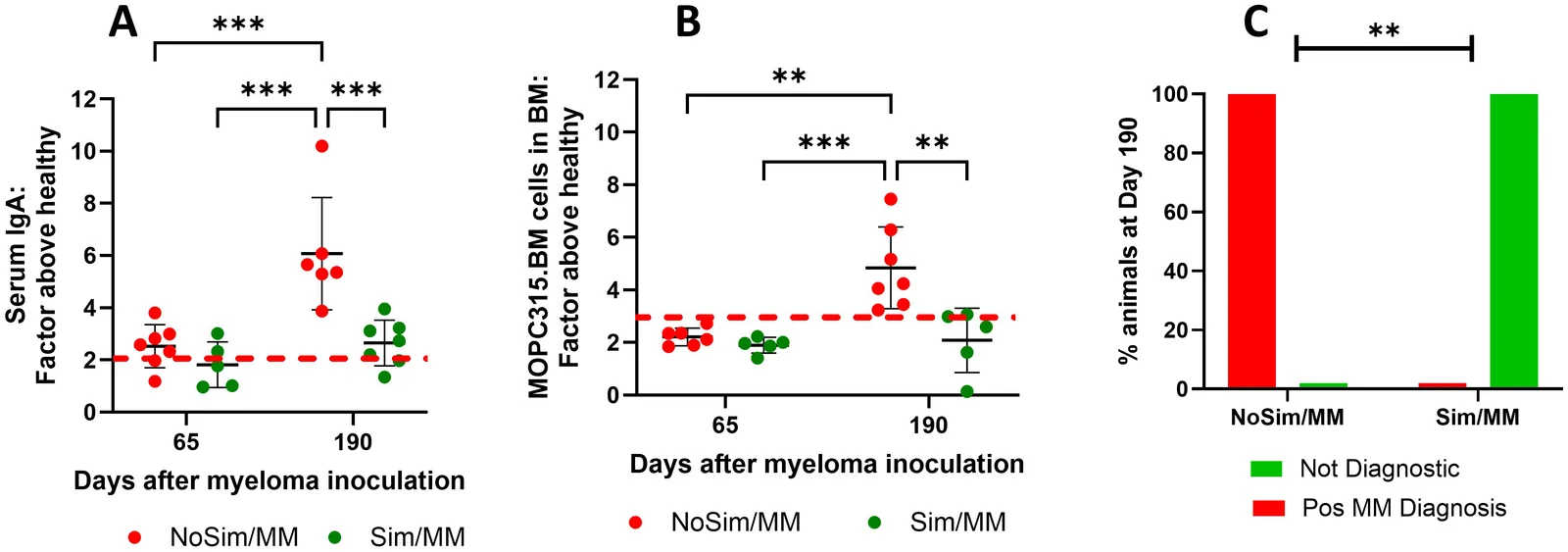
Influence of Simvastatin on designation of progressive myeloma. Increases in Serum IgA **(A)** and % MOPC315.BM **(B)** were calculated at Days 65 and 190. Increases in Serum IgA by a factor >2 and in % MOPC315.BM by a factor of >3.0 (see dashed red lines) were considered significant. Differences in factors between the groups were by two-way ANOVA with Bonferroni’s test. **(C)** % of animals with significant increases in both parameters at Day 190. Significant differences in positive and negative designation was assessed by two-way Fisher’s exact test. **p<0.001, ***p <0.0001.

Two important parameters that indicate progression towards clinically active MM are the levels of serum paraprotein and the myeloma plasma cell burden in the BM. To address the question whether SIM consumption affects MM progression, we tracked the increase in these parameters over time. Lending from the International Myeloma Foundation guidelines for designation of active myeloma, we defined a significant increase in serum paraprotein (in this case IgA) as being at least twice the average concentration in healthy mice, and a significant increase in myeloma tumor burden as three times that in healthy mice (in patients a doubling, from 5% to >10% in considered significant). **Figure 7** shows that at day 65 serum IgA and tumor burden were similar in myeloma-bearing mice whether or not they had received SIM (A, B). However, by day190, while both parameters had increased significantly in the NoSIM/MM group they had not changed in the SIM/MM group. Animals were then defined as having progressive myeloma if they were elevated in both parameters. By day 65, none of the animals had fulfilled this criteria. However, **Figure 7C** shows that by day 190, 100% of the NoSIM/MM mice compared to 0% of the SIM/MM mice could be defined as having progressive MM (P = 0.022, two-sided Fisher’s exact test).

## 4 Discussion

The initial goal of this study was to evaluate the effect of long-term use of Simvastatin on the incidence of Multiple Myeloma in a cohort representing the cross-cultural diversity of the Israeli population. The rationale for such a study was based on the established ability of SIM to disrupt the mevalonate pathway, a process critical for cholesterol biosynthesis (6). Cholesterol production is essential for the function of oncogenic proteins such as Ras and Rho GTPase, which transduce key proliferative signaling pathways for cancer cells such as MAPK/ERK and PI3K/Akt (11). In the BM, activation of the MAPK/ERK signaling pathway is essential for MM survival and proliferation (22) and the PI3K/Akt pathway promotes the translocation of glucose transporters to the cell membrane, ensuring efficient glucose import (23). Additionally, SIM can influence Ras-related signal transduction pathways (11, 24), and the drug appears to have complex effects on the immune system. It promotes the production of regulatory T cells (Tregs) (25), increases the expression of CD80 and CD86 molecules on monocytes, thereby enhancing T cell activation (9), and stimulates the secretion of inflammatory cytokines IL-1β and IL-12p70 (9).

Statins in general have wider effects on solid tumors of various types, although these effects seem to be cell origin and context dependent (26). This heterogeneity was recently emphasized in a systematic review of the effect of statin usage on cancer survival (27). Of 27 studies that reported reliable results, enhanced survival of patients with breast, lung, prostate, or head and neck cancer was positively correlated with statin use, as opposed to gastric or colorectal cancer, where no such correlation was found. There was insufficient data to conclude whether the use of statins was effective against cancers of the liver, pancreas, ovary, multiple myeloma, acute myeloid leukemia, glioblastoma, or uterus.

Regarding MM, Ponvilawan and colleagues reported in 2020 the results of a systematic review and meta-analysis of 10 criteria-eligible retrospective or case-controlled studies conducted in the USA, Europe, and Taiwan; their study found that statin usage was associated with a 20% reduction in the incidence of MM with a pooled Odds Ratio (OR) of 0.80 (28). When used in a therapeutic setting, it is noteworthy that an earlier study in six heavily pretreated MM patients found that high-dose SIM (15mg/Kg/day for 7 days followed by a rest period of 21 days in two 4-week cycles) stimulated bone turnover (29). Since the prescription of statins in Israel is similar to that of other developed countries (https://www.singlecare.com/blog/news/statin-statistics/), we carried out an epidemiological analysis of 15,204 Israeli citizens from different ethnic (genetic) backgrounds registered in the Leumit Health Fund database which, similar to the Ponvilawan study, found that SIM use during the 7-year exposure window, correlated with significant decrease in the incidence of MM in this cohort (OR of 0.798, **Table 1**).

To elucidate the mechanism(s) by which SIM may affect development of MM, we first demonstrated that at least *in vitro*, the drug has a cytotoxic effect on MM cell lines, although the cell lines differed in their drug sensitivity over several orders of magnitude, with the murine cell line MOPC315.BM being the most sensitive (**Figure 1**). In view of the known low bioavailability of SIM, it is noted that if the EC50 values reported here were translated to humans, only that for MOPC315.BM would be comparable with the projected nanomolar range of SIM in human plasma whereas those of human origin would require much larger doses.

While all three cell lines appear to die through apoptosis and not necrosis (**Figure 2**), the mechanisms leading to this pathway of cell death probably differ. SIM exposure caused a dramatic blockage of MOPC315.BM cells at the G2/M phase, while human U266 cells were blocked in G0/G1 and blockage of human RPMI-8226 cells occurred in G0/G1, S, and G2/M phases. This heterogeneity may reflect differences in kinetics of membrane biosynthesis, or different modes of action in cells from different genetic backgrounds and provides further support for the existence of distinct MM subpopulations (30, 31). Our results confirm previous reports on the cytotoxicity of SIM for MM cell lines (7, 32, 33) and consolidate the concept that the metabolic disturbances caused by SIM can directly affect several biological mechanisms in MM cells leading to their death (34).

Given the long-term, systemic use of SIM, it is important to investigate its influence on physiological systems, such as the immune system that affects tumor cell growth. SIM appears to have complex effects on the immune system (35). It increases monocyte expression of CD80 and CD86 molecules, thereby enhancing antigen presentation, T cell activation and the secretion of pro-inflammatory cytokines IL-1β and IL-12p70 (9), while several recent studies in immunocompromised animals demonstrate that treatment with SIM reduces the progression of some solid cancers (36, 37). To test the effects of SIM intake in a normal natural environment, we used our long-term “Early-to-Late” MM model (18) to investigate the influence of SIM on the anti-MM immune response in immunocompetent animals. This model tracks the changes in immune cell profiles and function and the immunosuppression that develops early in MM progression, as seen in human MM. The model highlights an early BM anti-myeloma T-cell mediated response which is ineffective and is replaced by progressive immunosuppression, dysfunction and exhaustion of CD8+ T cells. Now we demonstrate that treating MOPC315.BM myeloma-bearing mice every second day for 21 weeks with a human equivalent dose of SIM leads to a significant decrease in tumor cells in the BM (**Figure 3A**) and a significant enhancement of the BM anti-tumor immune response. These include a reversal of the decrease in CD4+ T cells seen in untreated animals with MM, which was accompanied by an elevation in CD8+ T cells mediated cytotoxicity of myeloma cells in SIM/MM mice (**Figure 6A**, p~0.08). These results suggest that SIM may help to overcome the immunosuppression that characterizes MM progression (**Figure 7**). The mechanism(s) driving these effects require further investigation, as SIM appears to promote the production of regulatory T cells (Tregs) (38, 39). Potentially, SIM may induce immunomodulatory effects that help create a more favorable environment for an immune response against MM cells.

A hint to at least one of these mechanisms can be seen in the results for the SIM/NoMM treatment group. These animals developed elevated levels of serum IgA by day 65 (p=0.0485) which were maintained at day190 (p=0.0285) (**Figure 3B**). This phenomenon was not limited to IgA, as total serum IgG levels were also significantly increased at Day 65 (p=0.013, **Figure 3C**). Although some of the immunomodulatory effects of SIM have been described (35, 40), these are the first data that provide direct evidence that SIM can modulate serum immunoglobulin levels.

An additional important question is whether long-term SIM intake affects MM progression? In the “Early-to-Late” MM model, animals have not yet developed clinical symptoms of active myeloma by day 190. But by combining two critical MM diagnostic parameters, namely significant elevation in both paraprotein and BM myeloma cell levels, we demonstrate that while not apparent in the early stages of the disease (day 65), SIM treatment significantly retarded myeloma progression by day 190 (**Figure 7**).

A limitation of this study is the depth to which we analyzed the mechanism of *in vitro* cell cytotoxicity induced by SIM exposure. Our goal at this stage of the project was to define the overall pathway of cell death. The details of the pathways probably alter, as suggested by our results, and deserve a separate study. Another limitation might be the depth of immune profiling performed on the *in vivo* model. Deep immune profiling on the ELMM has recently been published (18), and our goal in the present study was mainly to track changes in CD4+, CD8+ levels and CD8+ cytotoxic functionality. Our results indicate the potential benefits of long-term SIM exposure to the anti-myeloma immune response.

In summary, our results highlight two main points. First, that SIM can lead to myeloma cell death, presumably by mechanisms related to interference with lipid membrane synthesis so critical for tumor cell proliferation. Second, we show for the first time that long-term usage of Simvastatin enhances the T-cell mediated anti-tumor cell response in MM *in vivo*. Given that SIM is prescribed for the control of cholesterol production in adults of the same age group as patients developing MM disease, further understanding of the mechanism(s) underlying this immune modulation effect will help guide the repurposing and expanded use of SIM in MM therapy.

## Data Availability

The original contributions presented in the study are included in the article/**Supplementary Material**. Further inquiries can be directed to the corresponding authors.
