# Supplementary material for: Simvastatin as an immunomodulator and anti-myeloma agent – epidemiological, in vitro and murine model studies
Source: Front Immunol. 2026 Aug 19;17:1882291. doi: 10.3389/fimmu.2026.1882291 (PMC13534016; doi:10.3389/fimmu.2026.1882291)
Supplement: Supplementary file 1 [file Table1.docx]

**Supplementary Material**

### The Gating Strategy for the Types of Cells Studied

The hierarchical analysis of the flow cytometry data was performed using FlowJo V10.8.1software while applying a graduated gating strategy to identify and quantify specific cell populations, as detailed in Table S1. The gating process was carried out sequentially, with each subsequent step focusing the analysis on a more defined subpopulation, based on the expression profile of markers unique to each type, thus allowing for the isolation and precise definition of heterogeneous cell populations.

Table S1: Antibodies used for cell profiling

| Antibody | Fluorophore | Clone | Vendor |
| --- | --- | --- | --- |
| CD3 | APC/CY7 | 145-2011 | Biolegend |
| CD4 | BV605 | RM4-5 | Biolegend |
| CD8 | BV785 | 53-6.7 | Biolegend |
| CD3 | Unconjugated | 17A2 | Biolegend |
| CD28 | Unconjugated | 37.51 | Biolegend |
| B220 | PE CY7 | RA3-6B2 | Biolegend |
| CD138 | APC | REA104 | Milteny Biotech |
|  |  |  |  |

Table S2: Flow Cytometry Gating Strategy for identifying Bone Marrow lymphocytes and Myeloma cells

| *Cell Type* | *Gating Strategy* | | | | | |
| --- | --- | --- | --- | --- | --- | --- |
| T Helper Cells | CD3+ | CD4+ | CD8- |  |  |  |
| Cytotoxic T Cells | CD3+ | CD4- | CD8+ |  |  |  |
| MOPC315.BM | CD3- B220- | CD138+ |  |  |  |  |

Figure S1: Flow Cytometry Gating Strategy for identifying Bone Marrow lymphocytes
